# Opportunities to enhance antibiotic stewardship: colistin use and outcomes in a low-resource setting

**DOI:** 10.1093/jacamr/dlab169

**Published:** 2021-11-17

**Authors:** Muhammad S Moolla, Andrew Whitelaw, Eric H Decloedt, Coenraad F N Koegelenberg, Arifa Parker

**Affiliations:** 1 Division of General Medicine, Department of Medicine, Faculty of Medicine and Health Sciences, Stellenbosch University and Tygerberg Hospital, Cape Town, South Africa; 2 Division of Infectious Diseases, Department of Medicine, Faculty of Medicine and Health Sciences, Stellenbosch University and Tygerberg Hospital, Cape Town, South Africa; 3 Division of Medical Microbiology, Department of Pathology, Faculty of Medicine and Health Sciences, Stellenbosch University, Cape Town, South Africa; 4 National Health Laboratory Service, Tygerberg Hospital, Cape Town, South Africa; 5 Division of Clinical Pharmacology, Department of Medicine, Faculty of Medicine and Health Sciences, Stellenbosch University, Cape Town, South Africa; 6 Division of Pulmonology, Department of Medicine, Faculty of Medicine and Health Sciences, Stellenbosch University and Tygerberg Hospital, Cape Town, South Africa

## Abstract

**Background:**

Colistin use is increasing with the rise in MDR Gram-negative infections globally. Effective antibiotic stewardship is essential to preserve this antibiotic of last resort.

**Objectives:**

This study investigated stewardship and safety errors related to colistin use to identify opportunities for improvement.

**Patients and methods:**

A prospective descriptive study involving all patients 13 years and older treated with colistin at a tertiary hospital in Cape Town, South Africa, between August 2018 and June 2019. We collected clinical, laboratory and outcome data and assessed provided treatment for stewardship and safety errors.

**Results:**

We included 44 patients. Treatment errors were identified for 34 (77%) patients (median = 1), most commonly inadequate monitoring of renal function (*N *=* *16, 32%). We also identified no rational indication for colistin (*N *=* *9, 20%), loading dose error (*N *=* *12, 27%); maintenance dose error (*N *=* *10, 23%); no prior culture (*N *=* *11, 25%); and failure to de-escalate (2 of 9) or adjust dose to changes in renal function (6 of 15). All cause in-hospital mortality was 47%. Amongst survivors, median ICU stay was 6 days and hospital stay more than 30 days. Eight (18%) patients developed renal injury or failure during treatment. Three (7%) patients in this study were found to have colistin-resistant organisms including two prior to colistin exposure.

**Conclusions:**

This study has identified opportunities to enhance colistin stewardship and improve efficacy and safety of prescription. The appearance of colistin-resistant organisms reinforces the urgent need to ensure effective and appropriate use of colistin.

## Introduction

Gram-negative bacteria such as *Klebsiella pneumoniae*, *Acinetobacter* species, *Pseudomonas aeruginosa* and *Enterobacter* species cause infections including pneumonia, bloodstream infections, surgical site infections and meningitis. There has been an increase in MDR infections caused by these organisms, both globally^1^^,^^2^ and locally in South Africa.^3^^,^^4^ The threat caused by these organisms is so significant that they account for all the organisms listed as ‘Priority 1: Critical’ in the WHO priority pathogens list.^5^ When carbapenems, aminoglycosides and quinolones are ineffective, polymyxins serve as the last resort^6^^,^^7^ and their use is increasing.^8^

Colistin is a widely used polymyxin for its activity against MDR Gram-negative organisms.^9^ Newer β-lactamase inhibitor combinations such as ceftazidime/avibactam are not available in the state sector in South Africa due to cost. Colistin remains an important agent for MDR Gram-negative infections in low-to-middle-income countries (LMICs).^10^ Colistin is a concentration-dependent, bactericidal polypeptide antibiotic.^11^ Nephrotoxicity, followed by neurotoxicity, are its most common side effects. Both are dose-dependent and reversible on discontinuation of treatment,^1^^,^^12^^,^^13^ highlighting the need to determine optimal dosage and duration of treatment to minimize both toxicity and the emergence of resistance. This is particularly important for colistin as it has a narrow therapeutic window and large inter-patient pharmacokinetic variability.^14^ Therapeutic drug monitoring is recommended where available.^15^ While the true prevalence of colistin-resistant Gram-negative organisms in South Africa is not known, resistance to colistin has been reported both globally^16–18^ and locally.^19^

**Figure 1.**
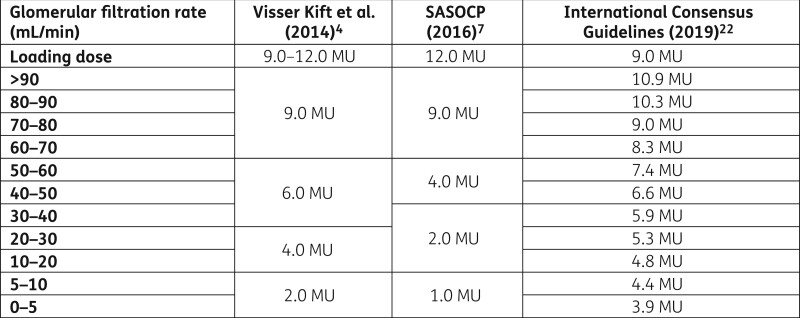
Summary of South African and international colistin dosing guidelines by total daily dose according to estimated glomerular filtration rate.

For years, incomplete knowledge of the pharmacokinetics and pharmacodynamics of colistin and outdated and conflicting literature about dosing have compromised its use in terms of both efficacy and safety.^20^^,^^21^ More recently local colistin guidelines were published by Visser Kift *et al*. in 2014^4^ and the South African Society of Clinical Pharmacy in 2016.^7^ International consensus guidelines were released in 2019.^22^ The discrepancies in the dosage recommendations between these guidelines as shown in Figure 1 may contribute to ongoing local prescriber uncertainty.

This study aimed to describe the current indications, antibiotic stewardship practices and outcomes related to colistin use at a tertiary academic hospital in Cape Town, South Africa, in order to identify antibiotic stewardship opportunities and optimize colistin prescribing practices and outcomes.

## Patients and methods

This was a prospective descriptive study at Tygerberg Hospital involving all consecutive patients 13 years and older treated with parenteral colistin from August 2018 to June 2019. Tygerberg Hospital is a 1300-bed tertiary academic hospital in Cape Town, South Africa. It is one of two academic referral centres in the city and renders a tertiary service to a population of approximately 3 million. The study was approved by the Stellenbosch University Health Research Ethics Committee (ref. no. S18/04/075) and written consent was obtained from participating patients.

Colistin is typically prescribed at Tygerberg Hospital for hospital-acquired pneumonia, soft tissue infections, septicaemia and intra-abdominal infections where a carbapenem-resistant, MDR organism is identified or a carbapenem has failed. Colistin is also used empirically during outbreaks of MDR organisms, based on locally appropriate protocols. Local colistin guidelines note a synergistic effect of combination therapy with a carbapenem^4^^,^^7^ if the MIC of the carbapenem in question is ≤8 mg/L. The choice of meropenem or imipenem in combination with colistin is based on which of the two has the lower MIC.^23^ Colistin use requires consultation and approval by a named infectious diseases (ID) and/or microbiology consultant before being issued by the pharmacy. Clinical pharmacy is a developing profession in South Africa and posts in the public sector for ward-based pharmacists are limited.^24^ The workload of procurement, preparation and dispensing of medication from the pharmacy does not allow adequate deployment of pharmacists from the pharmacy to the ward to provide a patient-orientated service at our institution. We have a clinical pharmacology service consisting of medically trained consultants and trainee registrars, but consultations are limited to challenging therapeutic cases and not routine review of all prescriptions. The service provided by clinical pharmacology extends to all disciplines and multiple therapeutic fields, including toxicology, and is not focused on antimicrobial stewardship.

Patients prescribed parenteral colistin during the study period were identified and referred by the hospital pharmacy, ID or microbiology departments for inclusion in the study. Inclusion criteria were (i) patients 13 years of age and older who were (ii) prescribed parenteral colistin and received at least one dose. Exclusion criteria were (i) patients less than 13 years of age or (ii) who had previously received a course of colistin.

Data were prospectively extracted from patient records and the National Health Laboratory Service (NHLS) TrakCare Web Results Viewer. Isolates were identified and susceptibility testing (apart from colistin) performed in the NHLS microbiology laboratory using the Vitek 2 automated identification system (bioMérieux, France). Colistin susceptibility was performed using broth microdilution in the NHLS reference laboratory in Johannesburg, Gauteng.^25^

The clinical syndrome was defined as the indication to initiate colistin documented in the notes by the treating physician. For the purpose of this study, the authors defined salvage therapy as the use of colistin after standard treatment options failed to produce an adequate clinical response in a patient, in the absence of a carbapenem-resistant isolate. The APACHE II scores were calculated using Calculate by QxMD Medical Software Inc.

Patients were followed up for a 30 day period after initiation of colistin, or until discharge or in-hospital death. We assessed the treatment course for a range of stewardship and safety errors. The decision to initiate colistin was evaluated for appropriateness by the study ID physician based on the clinical and microbiological data available at the time of initiation. Loading and maintenances doses were considered correct if they followed either of the two local guidelines available at the time of data collection. Other stewardship metrics that were assessed included not obtaining appropriate microbiological samples prior to initiating colistin therapy, use of combination antimicrobial therapy, monitoring of renal function, adjustment of colistin dose according to renal function and de-escalating antimicrobial therapy in response to culture results.

Acute kidney injury was classified according to the RIFLE criteria.^26^ Only the creatinine criteria could be applied as urine output was not consistently available for all patients. According to these criteria, risk represented a 1.5× increase in creatinine, injury a 2× increase and failure a 3× increase in creatinine.

Data analysis was done using SPSS (IBM). Descriptive numerical data with a normal distribution were described using means and standard deviation whereas non-normal data were described using median and range. A 95% CI was used to estimate effect sizes of the observed data.

### Availability of data and materials

The datasets used and/or analysed during the current study are available from the corresponding author on reasonable request.

## Results

### Recruitment

Forty-four patients were recruited. No patients were excluded or declined to participate in the study. All patients had complete follow up with complete data for analysis. Of the 44 patients, 32 patients were started on colistin in the ICU and 12 in the general wards.

### Baseline characteristics

Baseline characteristics are summarized in Table 1. The mean age of patients was 38 years and 61% (27/44) were male. More patients were admitted to the ICU compared with the general wards. There was an equal split between medical and surgical patients, where surgical patients included general surgical, burns, orthopaedics and gynaecology patients. The median (IQR) APACHE II score of the patients was 12 (7–18). Fourteen patients had acute kidney injury at the time of starting colistin, of whom four were receiving acute dialysis. Intermittent haemodialysis is used at our institution, with sustained low-efficiency dialysis used in patients unable to tolerate the former.

**Table 1.** Baseline characteristics at start of colistin therapy

Characteristic (*n *=* *44)	Value	(%)	[95% CI]
Age, years, mean	38.3		[33.7, 43.0]
Sex			
Male	27	(61.4)	[45.5, 75.3]
Female	17	(38.6)	[24.7, 54.5]
Level of care			
Intensive care	32	(72.7)	[56.9, 84.5]
General ward	12	(27.3)	[15.5, 43.1]
Discipline			
Medical	22	(50.0)	[34.8, 65.2]
Surgical	22	(50.0)	[34.8, 65.2]
APACHE II score, mean	12.8		[10.8, 14.8]
Albumin, g/L, mean	23.1		[21.0, 25.2]
Intubated	23	(52.3)	[36.9, 67.3]
Acute kidney injury	14	(31.8)	[19.1, 47.7]
Acute dialysis	4	(9.1)	[3.0, 22.6]
HIV positive	8	(18.2)	[8.7, 33.2]
Time since admission to start of therapy, days (IQR)	14		(9–20)

### Indications for treatment

The most common clinical syndromes being treated were hospital-acquired pneumonia (*n *=* *16; 36.3%) and soft tissue infections (*n *=* *14; 31.8%). Eight patients (18.1%) were treated for intra-abdominal infection and six (13.6%) for bloodstream infections without a documented underlying source.

Based on microbiology results available at the time of colistin initiation, most patients (*n *=* *27; 61.4%) were treated with colistin as directed therapy. Twelve patients (27.3%) were treated empirically while five (11.4%) were given colistin as part of salvage therapy.

In just over half of patients, the infecting organism was isolated from blood culture (*n *=* *23; 52.3%). Respiratory samples, primarily tracheal aspirates, and tissue cultures were positive in 29.5% and 27.3% of patients respectively. Five (11.4%) patients had organisms identified on superficial swabs, three (6.8%) on urine culture and three (6.8%) on central line tip.

The most common Gram-negative organism found was *Acinetobacter baumannii* in 27 (73.0%) patients. *K. pneumoniae* was found in 9 (24.3%) patients and *P. aeruginosa* in 1 (2.7%). Colistin MICs were available in 28 patients. The median MIC of these organisms was 0.75 mg/L (IQR: 0.5–1). One organism, a *K. pneumoniae* isolate, had an MIC of 2 mg/L. Two isolates of *A. baumannii* from surgical patients with no prior colistin exposure were found to be resistant to colistin.

### Antibiotic stewardship errors

Antibiotic stewardship errors were identified for 18 (40.9%) of the patients treated with colistin as shown in Table 2. Despite the colistin restriction on prescribing policy, nine (20.5%) cases were considered to not have an appropriate indication based on the clinical picture and available culture results. This was similar across level of care and discipline.

**Table 2.** Stewardship and safety measures

	Overall,		ICU,		General,		Medical,		Surgical,	
	*n *=* *44		*n *=* *32		*n *=* *12		*n *=* *22		*n *=* *22	
Antibiotic stewardship errors	18	(40.9)	14	(43.8)	4	(33.3)	7	(31.8)	11	(50.0)
Inappropriate choice	11	(25.0)	7	(21.8)	2	(16.6)	6	(27.2)	3	(13.6)
No prior culture	11	(25.0)	7	(21.9)	4	(33.3)	0	(0.0)	11	(50.0)
Not treated with combination therapy	11	(25.0)	6	(18.8)	5	(41.7)	5	(22.7)	6	(27.2)
Failed to de-escalate	2	of 9	1	of 8	1	of 1	1	of 5	1	of 4
Safety errors	29	(65.9)	18	(56.3)	11	(91.7)	11	(0.50)	18	(81.8)
Loading dose error	12	(27.3)	6	(18.8)	6	(50.0)	3	(13.6)	9	(40.9)
Incorrect loading dose	4	(9.1)	3	(9.4)	1	(8.3)	0	(0.0)	4	(18.2)
Loading dose not prescribed	8	(18.2)	3	(9.4)	5	(41.7)	3	(13.6)	5	(22.7)
Maintenance dose error	10	(22.7)	8	(25.0)	2	(16.7)	5	(22.7)	5	(22.7)
Incorrect maintenance dose	7	(15.9)	3	(9.4)	0	(0.0)	2	(9.1)	1	(4.5)
Initial maintenance dose not according to GFR	3	(6.8)	5	(15.6)	2	(16.7)	3	(13.6)	4	(18.2)
Not dosed according to either local guideline	16	(36.4)	10	(31.3)	6	(50.0)	5	(22.7)	11	(50.0)
Renal function not monitored	16	(36.4)	6	(18.8)	10	(83.3)	5	(22.7)	11	(50.0)
Maintenance dose not adjusted after change in GFR	6	of 15	6	of 13	0	of 2	3	of 8	3	of 7
Total errors per patient, median (IQR)	1	(1–2)	1	(0–2)	2	(1–3)	1	(0–2)	2	(1–3)
0	10	(22.7)	9	(28.1)	1	(8.3)	7	(31.8)	3	(13.6)
1	16	(36.4)	12	(37.5)	4	(33.3)	9	(40.9)	7	(31.8)
2	2	(20.5)	6	(18.6)	3	(25.0)	4	(18.2)	5	(22.7)
3	4	(9.1)	3	(9.4)	1	(8.3)	2	(9.1)	2	(9.1)
4	5	(11.4)	2	(6.3)	3	(25.0)	0	(0.0)	5	(22.7)

Values are shown as *n* (%) unless stated otherwise.

GFR, glomerular filtration rate.

No culture was performed prior to starting colistin in 11 (25.0%) patients.

In 11/44 (25.0%) scripts, no combination therapy was prescribed. This included 7/27 (25.9%) patients with *A. baumannii*, 2/9 (22.2%) with *K. pneumoniae*, the single patient with *P. aeruginosa* and 1/7 (14.3%) without an identified organism. The most likely antimicrobials to be empirically co-prescribed with colistin were meropenem (65.9%) and imipenem (9.1%).

In nine (20.5%) patients, the results of bacterial culture and susceptibility allowed for de-escalation of antimicrobial therapy. This did not occur for two of these patients.

### Safety errors

Safety errors were identified for 29 (65.9%) patients. Twenty-eight patients received colistin doses according to one of the South African guidelines. The dosages prescribed for 16 patients did not comply with either of the local guidelines available at the time. Errors with the loading dose were found in 12 (27.3%) patients. These errors were more likely to occur in the general wards (50.0%) compared with intensive care (18.8%) and in surgical patients (40.9%) compared with medical patients (13.6%), but the difference did not reach statistical significance. Errors included not prescribing a loading dose (18.2%) and prescribing an incorrect loading dose (9.1%). Ten (22.7%) scripts had maintenance dose errors including seven (15.9%) where the dose was incorrect and three (6.8%) where the dose had not been adjusted according to the renal function.

Renal function was not monitored during the course of colistin therapy in 16 (36.4%) patients. This was more often found in the general wards (83.3%) and for surgical patients (50.0%). Where monitored, 15 (34.1%) patients had a change in renal function necessitating an adjustment of the colistin maintenance dose. In 6 of these 15 cases, the dose was not adjusted.

**Figure 2.**
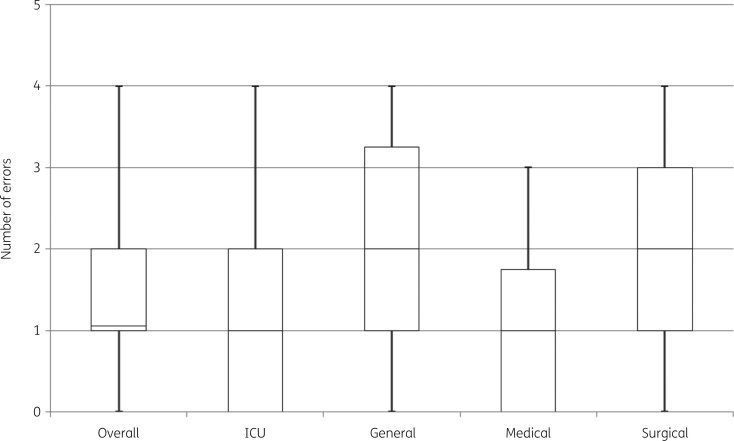
Box-and-whisker plot of the number of stewardship and safety errors found overall, by level of care and by discipline. Central line indicates the median number of errors found, box indicates the IQR and whiskers indicates the range.

Overall, errors of any type were identified for 34 (77.3%) of the patients treated with colistin with a median of 1 (IQR: 1–2) prescribing error per patient as shown in Table 2. The number of errors in general ward and surgical patients were similar; the median was two (Figure 2). The maximum number of errors found for a patient was four, in five (11.4%) patients.

### Outcomes

Thirty-day all-cause mortality was 47.7% (95% CI: 32.7, 63.1). Admission APACHE II scores were significantly higher amongst patients who died (median [IQR]=16 [11–21]) compared with those who survived (median [IQR]=8 [6–14]) (*P *=* *0.0003). Higher mortality was observed amongst medical (54.5% [95% CI: 32.6, 74.9]) compared with surgical patients (40.9% [95% CI: 21.5, 63.3]) and amongst those treated according to either of the South African guidelines (53.6% [95% CI: 34.2, 72.0]) compared with those dosed incorrectly (37.5% [95% CI: 16.3, 64.13]), but this was not statistically significant.

Amongst patients that survived, median length of ICU stay was 6 days (IQR: 0–15) with some patients remaining in ICU for more than 30 days. Median length of hospital stay for all patients who survived was more than 30 days (IQR: 19 to >30).

The all-cause acute kidney injury rate was 18.2% [95% CI: 8.7, 33.3]. This consisted of five patients (11.4%) who developed risk, six (13.6%) injury and two (4.5%) failure.^26^ The nephrotoxicity rate was higher amongst surgical (27.3% [95% CI: 11.6, 50.5]) compared with medical patients (9.1% [95% CI: 1.6, 30.6]) and between those treated according to either South African guideline (25.0% [95% CI: 11.4, 45.2]) compared with those treated incorrectly (6.3% [95% CI: 0.3, 32.3]), although none of these differences reached statistical significance. Similar rates were observed amongst ICU and general ward patients.

Two patients continued to grow organisms from sterile sites while on treatment with colistin and six after treatment with colistin. All except one of these patients were treated according to one of the South African guidelines. This last patient, a surgical patient in ICU not treated according to the guideline, cultured a colistin-resistant *A. baumannii*.

## Discussion

This study has shown a large number of stewardship and safety errors with the use of colistin. The errors occur at all points in the treatment course, from the decision to initiate therapy through dosing to monitoring and potential de-escalation. The study also showed a very high risk of death amongst patients started on colistin. These findings indicate that clinicians require a greater degree of guidance and support when prescribing colistin and that more research is required to optimize its use.

Our findings indicate that antibiotic stewardship principles and programmes are not effectively followed and enforced and need to be strengthened for colistin in particular, and antimicrobials in general. Errors in dosing and monitoring also point to a lack of knowledge amongst clinicians regarding the effective and safe use of colistin. This may be due to a lack of familiarity with the drug compared with other antimicrobials. Other potential explanations include conflicting dosages and units of measurement in guidelines and manufacturer inserts, lack of awareness of or difficulty accessing published guidelines, and outdated formularies.^20^^,^^21^ These issues may also, in part, explain the high mortality rate seen in this study.

Evidence suggest that only 30%–40% of patients receiving currently recommended colistin dosages are expected to reach the target average steady-state plasma concentration (c_ss_) of 2 mg/L and 80% reach a c_ss_ of 1 mg/L.^22^ Therefore, even patients who were treated correctly may have failed to achieve therapeutic colistin concentrations. This is especially true of patients with normal renal function. Concentrations reached at target sites, especially the lung, can be significantly lower resulting in inadequate therapy. For this reason, CLSI has recently opted to eliminate the ‘susceptible’ interpretive category for colistin, instead assigning ‘intermediate’ to any MIC ≤2 mg/L,^27^ illustrating how difficult colistin dosing can be, even for experienced clinicians. Colistin therapeutic drug monitoring is also not available in our setting.

One multicentre retrospective record review involving private sector hospitals in South Africa had similar findings, with adherence to local guidelines at best 48.2%.^28^ Inappropriate use of antibiotics in general is also known to be high.^29^ While initial reports showed nephrotoxicity rates with colistin of almost 50%, our findings are comparable to more recent international reports with lower rates of 10% to 30%.^4^^,^^15^^,^^30^

We are not aware of any local outcome data for colistin. Internationally, mortality rates vary broadly between 30% and 70% but studies are heterogenous in terms of population, treatment regimen and outcomes.^31–34^ The finding that patients treated according to guidelines had worse outcomes than those treated incorrectly was unexpected. Several factors may explain this. This was a prospective study and any treatment errors were corrected when discovered, thus many patients in the latter group were ultimately treated correctly. Patients treated according to the guidelines were more likely to be ICU or medical patients, two groups which themselves had worse outcomes. Confounding may therefore have played a role. Lastly, the small patient numbers in the subgroups means that chance may have played a role in this outcome as the differences, although large, were not statistically significant.

### Limitations

Colistin was not indicated in all patients, while 30 day all-cause mortality was considered a crude measure of this secondary outcome since it may reflect many factors other than the effectiveness of colistin therapy. Response to treatment may have been a better method to determine the number of patients where colistin therapy failed. However, this was beyond the scope and resources available in this study. Similarly, acute kidney injury can more accurately be determined using biological markers, while the urine output component of the RIFLE criteria could not be included in analysis. As this was a descriptive study, renal function and culture results could only be checked opportunistically when requested by the attending clinician and not according to a protocol. This resulted in incomplete data for these outcomes. Data regarding the adequacy of source control procedures were not available. Data regarding pre-authorization of colistin by an ID and/or microbiology consultant prior to pharmacy supply were not collected in this study. This is an important antimicrobial stewardship intervention and rates of pre-authorization should be measured in future studies.

### Recommendations

Current colistin guidelines are based on international pharmacokinetic data and have not been tested in randomized control trials.^14^^,^^35^ There is an urgent need for colistin pharmacokinetic studies in all patient populations including special populations such as the critically ill and obese. Studies generating pharmacokinetic data are needed to optimize future guidelines.

More urgently, there is a need for antibiotic stewardship activities to be implemented more stringently, consistently and effectively in the clinical context. Following this study, our hospital implemented new protocols for the use of colistin. We have adapted the new international guidelines^22^ for use at our institution. We also began collaborating internationally to develop expertise in colistin pharmacokinetic measurement and dose optimization in special populations. The efficacy of these changes at our institution is still to be investigated, but the implementation of an antimicrobial stewardship programme for colistin in another lower-middle-income country showed significant improvement across all measures.^36^ Other facilities are urged to similarly audit colistin use to inform antibiotic stewardship and quality improvement processes. Education and training of healthcare workers on the safe and effective use of colistin is required and there are opportunities for the ID and microbiology teams to remain more involved, though care should be taken not to delay appropriate antibiotic initiation. Expansion of the clinical pharmacy service would allow many of the antimicrobial stewardship and safety errors identified in this study to be detected and corrected, but remains a challenge due to resource constraints in LMICs.^24^ Making therapeutic drug monitoring for colistin available locally will help to overcome current challenges with dosing and raise awareness of issues around monitoring. Consideration should also be given to a switch to polymyxin B, which has the benefits of being administered in active form and not requiring renal dose adjustments, making it more prescriber-friendly.^22^^,^^37–39^ However, alternatives remain costly in low-resource settings and challenging to procure.

### Conclusions

Colistin has acquired the title of antibiotic of last resort and is unlikely to be replaced soon. The spectre of resistance looms large and efforts towards elucidating its pharmacological properties must be matched by equal efforts to strengthen antibiotic stewardship programmes and ensure that colistin remains an effective option long into the future.

## References

[1] Biswas S , Brunel JM, Dubus JC et al Colistin: an update on the antibiotic of the 21st century. Expert Rev Anti Infect Ther 2012; 10: 917–34. 23030331 10.1586/eri.12.78

[2] Kanj SS , Kanafani ZA. Current concepts in antimicrobial therapy against resistant gram-negative organisms: extended-spectrum β-lactamase-producing Enterobacteriaceae, carbapenem-resistant Enterobacteriaceae, and multidrug-resistant *Pseudomonas aeruginosa*. Mayo Clin Proc 2011; 86: 250–9. 21364117 10.4065/mcp.2010.0674 PMC3046948

[3] Bamford C , Bonorchis K, Elliott E et al Antimicrobial susceptibility patterns of selected bacteraemic isolates from public sector hospitals in South Africa. S Afr J Epidemiol Infect 2009; 24: 28–30.

[4] Visser Kift E , Maartens G, Bamford C. Systematic review of the evidence for rational dosing of colistin. S Afr Med J 2014; 104: 183–6. 24897820

[5] WHO. Global Priority List of Antibiotic-Resistant Bacteria to Guide Research, Discovery and Development of New Antibiotics. https://www.who.int/medicines/publications/WHO-PPL-Short_Summary_25Feb-ET_NM_WHO.pdf.

[6] Falagas ME , Rafailidis PI, Ioannidou E et al Colistin therapy for microbiologically documented multidrug-resistant Gram-negative bacterial infections: a retrospective cohort study of 258 patients. Int J Antimicrob Agents 2010; 35: 194–9. 20006471 10.1016/j.ijantimicag.2009.10.005

[7] Labuschagne Q , Schellack N, Gous A et al COLISTIN: adult and paediatric guideline for South Africa, 2016. S Afr J Infect Dis 2016; 31: 3–7.

[8] Van Boeckel TP , Gandra S, Ashok A et al Global antibiotic consumption 2000 to 2010: an analysis of national pharmaceutical sales data. Lancet Infect Dis 2014; 14: 742–50. 25022435 10.1016/S1473-3099(14)70780-7

[9] Yahav D , Farbman L, Leibovici L et al New lessons on an old antibiotic. Clin Microbiol Infect 2012; 18: 18–29. 22168320 10.1111/j.1469-0691.2011.03734.x

[10] Karaiskos I , Lagou S, Pontikis K et al The “old” and the “new” antibiotics for MDR Gram-negative pathogens: for whom, when, and how. Front Public Heal 2019; 7: 151. 10.3389/fpubh.2019.00151 PMC6581067 31245348

[11] Poirel L , Jayol A, Nordmann P. Polymyxins: antibacterial activity, susceptibility testing and resistance mechanisms encoded by plasmid or chromosomes. Clin Microbiol Rev 2017; 30: 557–96. 28275006 10.1128/CMR.00064-16 PMC5355641

[12] Pike M , Saltiel E. Colistin- and polymyxin-induced nephrotoxicity: focus on literature utilizing the RIFLE classification scheme of acute kidney injury. J Pharm Pract 2014; 27: 554–61. 25237156 10.1177/0897190014546116

[13] Falagas ME , Kasiakou SK. Toxicity of polymyxins: a systematic review of the evidence from old and recent studies. Crit Care 2006; 10: R27. 16507149 10.1186/cc3995 PMC1550802

[14] Nation RL , Garonzik SM, Thamlikitkul V et al Dosing guidance for intravenous colistin in critically-ill patients. Clin Infect Dis 2017; 64: 565–71. 28011614 10.1093/cid/ciw839 PMC5850520

[15] Vicari G , Bauer SR, Neuner EA et al Association between colistin dose and microbiologic outcomes in patients with multidrug-resistant Gram-negative bacteremia. Clin Infect Dis 2013; 56: 398–404. 23090926 10.1093/cid/cis909

[16] Jayol A , Poirel L, Brink A et al Resistance to colistin associated with a single amino acid change in protein PmrB among *Klebsiella pneumoniae* isolates of worldwide origin. Antimicrob Agents Chemother 2014; 58: 4762–6. 24914122 10.1128/AAC.00084-14 PMC4136042

[17] Al-Tawfiq JA , Laxminarayan R, Mendelson M. How should we respond to the emergence of plasmid-mediated colistin resistance in humans and animals? Int J Infect Dis 2017; 54: 77–84. 27915108 10.1016/j.ijid.2016.11.415

[18] Marchaim D , Chopra T, Pogue JM et al Outbreak of colistin-resistant, carbapenem-resistant *Klebsiella pneumoniae* in Metropolitan Detroit, Michigan. Antimicrob Agents Chemother 2011; 55: 593–9. 21115786 10.1128/AAC.01020-10 PMC3028794

[19] Coetzee J , Corocoran C, Prentice E et al Emergence of plasmid-mediated colistin resistance (MCR-1) among *Escherichia coli* isolated from South African patients. S Afr Med J 2016; 106: 35–6. 27138657 10.7196/SAMJ.2016.v106i5.10710

[20] Nation RL , Li J, Cars O et al Framework for optimisation of the clinical use of colistin and polymyxin B: the Prato polymyxin consensus. Lancet Infect Dis 2015; 15: 225–34. 25459221 10.1016/S1473-3099(14)70850-3

[21] Ortwine JK , Kay KS, Li J et al Colistin: understanding and applying recent pharmacokinetic advances. Pharmacotherapy 2015; 35: 11–6. 25187500 10.1002/phar.1484

[22] Tsuji BT , Pogue JM, Zavascki AP et al International Consensus Guidelines for the optimal use of the polymyxins: endorsed by the American College of Clinical Pharmacy (ACCP), European Society of Clinical Microbiology and Infectious Diseases (ESCMID), Infectious Diseases Society of America (IDSA). Pharmacotherapy 2019; 39: 10–39. 30710469 10.1002/phar.2209 PMC7437259

[23] Daikos GL , Tsaousi S, Tzouvelekis LS et al Carbapenemase-producing *Klebsiella pneumoniae* bloodstream infections: lowering mortality by antibiotic combination schemes and the role of carbapenems. Antimicrob Agents Chemother 2014; 58: 2322–8. 24514083 10.1128/AAC.02166-13 PMC4023796

[24] Bronkhorst E , Gous AGS, Schellack N. Practice guidelines for clinical pharmacists in middle to low income countries. Front Pharmacol 2020; 11: 978. 32695002 10.3389/fphar.2020.00978 PMC7338713

[25] CLSI. *Performance Standards for Antimicrobial Susceptibility testing—Thirty-First Edition: M100*. 2021.

[26] Kellum JA , Bellomo R, Ronco C. Definition and classification of acute kidney injury. Nephron Clin Pract 2008; 109: 182–7. 10.1159/000142926 18802365

[27] Satlin MJ , Lewis JS, Weinstein MP et al Clinical and Laboratory Standards Institute and European Committee on Antimicrobial Susceptibility Testing position statements on polymyxin B and colistin clinical breakpoints. Clin Infect Dis 2020; 71: e523–9. 32052041 10.1093/cid/ciaa121

[28] Messina AP , Brink AJ, Richards GA et al Opportunities to optimise colistin stewardship in hospitalised patients in South Africa: results of a multisite utilisation audit. S Afr Med J 2017; 108: 28–32. 29262975 10.7196/SAMJ.2017.v108i1.12561

[29] Paruk F , Richards G, Scribante J et al Antibiotic prescription practices and their relationship to outcome in South Africa: findings of the prevalence of infection in South African intensive care units (PISA) study. S Afr Med J 2012; 102: 613–6. 22748439 10.7196/samj.5833

[30] Pogue JM , Lee J, Marchaim D et al Incidence of and risk factors for colistin-associated nephrotoxicity in a large academic health system. Clin Infect Dis 2011; 53: 879–84. 21900484 10.1093/cid/cir611

[31] Tanita MT , Carrilho CMD, Garcia JP et al Parenteral colistin for the treatment of severe infections: a single center experience. Rev Bras Ter Intensiva 2013; 25: 297–305. 24553511 10.5935/0103-507X.20130051 PMC4031873

[32] Balkhair A , Al-Muharrmi Z, Al'Adawi B et al Prevalence and 30-day all-cause mortality of carbapenem-and colistin-resistant bacteraemia caused by *Acinetobacter baumannii*, *Pseudomonas aeruginosa*, and *Klebsiella pneumoniae*: description of a decade-long trend. Int J Infect Dis 2019; 85: 10–5. 31100418 10.1016/j.ijid.2019.05.004

[33] Benattar YD , Omar M, Zusman O et al The effectiveness and safety of high-dose colistin: prospective cohort study. Clin Infect Dis 2016; 63: 1605–12. 27794023 10.1093/cid/ciw684

[34] Sorlí L , Luque S, Segura C et al Impact of colistin plasma levels on the clinical outcome of patients with infections caused by extremely drug-resistant *Pseudomonas aeruginosa*. BMC Infect Dis 2017; 17: 11. 28056821 10.1186/s12879-016-2117-7 PMC5217330

[35] Garonzik SM , Li J, Thamlikitkul V et al Population pharmacokinetics of colistin methanesulfonate and formed colistin in critically ill patients from a multicenter study provide dosing suggestions for various categories of patients. Antimicrob Agents Chemother 2011; 55: 3284–94. 21555763 10.1128/AAC.01733-10 PMC3122440

[36] Sathyapalan DT , James J, Sudhir S et al Antimicrobial stewardship and its impact on the changing epidemiology of polymyxin use in a South Indian healthcare setting. Antibiotics 2021; 10: 470. 33918994 10.3390/antibiotics10050470 PMC8142974

[37] Sandri AM , Landersdorfer CB, Jacob J et al Population pharmacokinetics of intravenous polymyxin B in critically ill patients: implications for selection of dosage regimens. Clin Infect Dis 2013; 57: 524–31. 23697744 10.1093/cid/cit334

[38] Kwa A , Kasiakou SK, Tam VH et al Polymyxin B: similarities to and differences from colistin (polymyxin E). Expert Rev Anti Infect Ther 2007; 5: 811–21. 17914915 10.1586/14787210.5.5.811

[39] Nation RL , Velkov T, Li J. Colistin and polymyxin B: peas in a pod, or chalk and cheese? Clin Infect Dis 2014; 59: 88–94. 24700659 10.1093/cid/ciu213 PMC4305129

